# Contribution of extended family history in assessment of risk for breast and colon cancer

**DOI:** 10.1186/s12875-016-0521-0

**Published:** 2016-09-01

**Authors:** Benjamin L. Solomon, Todd Whitman, Marie E. Wood

**Affiliations:** 1 University of Vermont Medical Center, 111 Colchester Ave, Burlington, VT 05401 USA; 2 Champlain Valley Physicians Hospital, 75 Beekman St, Plattsburgh, NY 12901 USA

**Keywords:** Colon cancer, Breast cancer, Family history, Cancer screening, Genetic screening, Cancer risk, Risk assessment

## Abstract

**Background:**

Family history is important for identifying candidates for high risk cancer screening and referral for genetic counseling. We sought to determine the percentage of individuals who would be eligible for high risk cancer screening or genetic referral and testing if family history includes an extended (vs limited) family history.

**Methods:**

Family histories were obtained from 626 women at UVMMC associated mammography centers from 2001 to 2002. ACS guidelines were used to determine eligibility for high risk breast or colon cancer screening. Eligibility for referral for genetic counseling for hereditary breast and colon cancer was determined using the Referral Screening Tool and Amsterdam II screening criteria, respectively. All family histories were assessed for eligibility by a limited history (first degree relatives only) and extended history (first and second degree relatives).

**Results:**

Four hundred ninety-nine histories were eligible for review. 18/282 (3.6 %) and 62/123 (12 %) individuals met criteria for high risk breast and colon cancer screening, respectively. 13/18 (72 %) in the high risk breast cancer screening group and 12/62 (19 %) in the high risk colon cancer screening group met criteria based upon an extended family history. 9/282 (1.8 %) and 31/123 (6.2 %) individuals met criteria for genetic counseling referral and testing for breast and colon cancer, respectively. 2/9 (22 %) of individuals in the genetic breast cancer screening group and 21/31 (68 %) individuals in the genetic colon cancer screening group met criteria based upon extended family history.

**Conclusions:**

This is one of the first studies to suggest that first degree family history alone is not adequate for identification of candidates for high risk screening and referral for genetic counseling for hereditary breast and colon cancer syndromes. A larger population is needed to further validate this data.

**Electronic supplementary material:**

The online version of this article (doi:10.1186/s12875-016-0521-0) contains supplementary material, which is available to authorized users.

## Background

A family history of colorectal or breast cancer may significantly impact screening and management strategies for patients. Individuals with a positive family history of colon cancer may be candidates for earlier initiation of colonoscopy. Individuals with a positive family history of breast cancer may be candidates for screening breast MRI or be eligible for prevention strategies (i.e., chemoprophylaxis and/or surgical prophylaxis including bilateral mastectomy and bilateral salpingo-oophorectomy) [1]. Furthermore, individuals with a family history suggesting a hereditary cancer syndrome, such as Lynch syndrome or hereditary breast and ovarian cancer syndrome, should be referred for genetic counseling and/or genetic testing. Multiple guidelines for breast and colon cancer high risk screening and referral for genetic counseling and genetic testing require knowledge of first, second and occasionally third degree relatives [2–5].

Although obtaining a family history is a staple of primary care management, there are significant barriers to obtaining a complete and accurate family history. These barriers include time restraints, patient knowledge and lack of perceived importance by healthcare provider [6, 7]. A key barrier is the lack of standardization of required information. The gold standard family history is a five generation pedigree used in medical genetics and genetic counseling [8]. Recently, the American Society of Clinical Oncology (ASCO) has suggested that a minimum family history for oncology patients should include first and second degree relatives, type of cancer, age at cancer diagnosis and lineage (maternal and/or paternal) [9].

Providers commonly obtain information from first degree relatives but less commonly obtain a more extended family history or age at cancer diagnosis [10, 11]. The current cross-sectional study was undertaken to determine the value of an extended (first and second degree) family history compared to a limited (first degree only) family history in determining eligibility for high risk screening and referral for genetic counseling for breast or colon cancer.

## Methods

Women presenting for breast cancer screening at one of several mammography facilities associated with the University of Vermont Medical Center (UVMMC) were given the opportunity to complete and return a family history questionnaire. All women were invited to participate including those for asymptomatic screening and symptomatic referrals. The average age was 63 years and 100 % were female. A consent form was attached to the front of the questionnaire providing a description of the study and requesting permission to use information for this study. Permission was also obtained to contact patients for clarification of information on the questionnaire if necessary. Questionnaires were distributed between May 2001 and May 2002. The study was approved by the UVMMC IRB.

### Questionnaire

Information on all related family members was obtained via questionnaire (see Additional file 1) including gender, lineage (maternal or paternal), relatedness (sibling, parents, aunts, uncles, etc.), primary cancer and age at diagnosis. This questionnaire has been developed and pilot tested in the Familial Cancer Program at the University of Vermont Cancer Center.

### Screening guidelines and risk models utilized

Breast Cancer Screening Guidelines and Models for Risk Assessment: The American Cancer Society (ACS) and National Comprehensive Cancer Network (NCCN) consider women as high risk with a greater than 20 % lifetime risk of breast cancer due to family history and recommend adding annual screening breast magnetic resonance imaging (MRI) to their annual mammogram for breast cancer screening [3, 12]. We calculated risk of developing breast cancer based on family history using the Claus Model [13]. This model uses first and second degree family history and age at cancer diagnosis to determine risk. While other models exist (Gail, Tyrer-Cuzick, etc.), the Claus model is the only model which calculates risk solely based on family history. Therefore, the Claus model would not confound the results with factors affecting risk beyond family history.

There are several guidelines regarding referral for genetic counseling and testing for hereditary breast cancer including guidelines from American College of Obstetrics and Gynecology (ACoSOG), the National Comprehensive Cancer Network (NCCN) and the US Preventative Service Task Force (USPSTF) [4, 14, 15]. We chose to use the USPSTF guidelines as they include an extended family history and are easy to administer. The USPSTF recommends consideration of several validated screening tools including the Referral Screening Tool (RST). The RST is one of the easiest to use and therefore appropriate for busy primary care providers [16]. The RST includes first or second degree relatives 50 years or younger with history of breast cancer, any first or second degree relative with ovarian cancer, male breast cancer or greater than one family member on the same side of the family over the age of 50 with breast cancer. Patients should be referred for genetic counseling if they meet two or more of these criteria.

Colorectal Cancer Screening Guidelines and Models for Risk Assessment: Several guidelines exist for colon cancer screening; however, only the ACS includes extended family history in recommendations for high risk individuals. These recommendations suggest that screening begin at age 40 for individuals with any first degree or two second degree relatives at any age with a history of colorectal cancer [17].

Guidelines for referral for genetic counseling or testing for hereditary colon cancer for individuals with no personal history of colorectal cancer have not been developed at this time. However, Hampel at al. suggest that individuals with a lifetime relative risk (RR) ratio of greater than 2.0 be referred for genetic counseling [18]. This criteria can be met using the Amsterdam II criteria [19]. Individuals meet criteria if they have one or more of the following: three or more first or second degree relatives with any HNPCC associated cancers, one relative with two or more HNPCC associated cancers or one first degree relative with colorectal cancer less than 50 years old.

### Statistical analysis

All questionnaires were transcribed into a Microsoft Access database. Each study participant was assigned a unique, random identification number to allow depersonalization of data. The data was queried for individuals with at least one relative with a primary cancer excluding non-melanomatous skin cancers. This data was then queried to determine individuals who met specific guideline criteria based on their first degree family history followed by individuals who met criteria solely based on a first degree family history.

## Results

### Study population

**Fig. 1:**
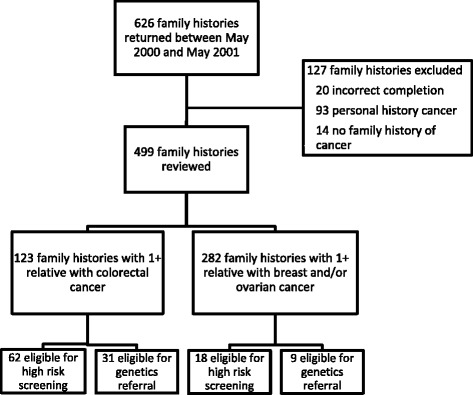
Consort diagram. Sixty-six family histories have both colorectal cancer and breast and/or ovarian family history and were counted in both groups

Six hundred twenty-six family history questionnaires were returned between May 2001 and May 2002. Questionnaires were excluded from this analysis if they were not completed correctly, the participant completing the questionnaire had a history of cancer or there was no cancer in the family (see Fig. 1 for details). Four hundred ninety-nine met inclusion criteria and were reviewed. 359 (71.9 %) participants had at least one family member with breast or colon cancer; 282 (56.5 %) had at least one family member with breast cancer and 123 (24.6 %) had at least one family member with colorectal cancer. 66 (13.2 %) of participants had member(s) of the family with breast and colorectal cancer.

### Identification of candidates for high risk breast cancer screening or referral for hereditary breast cancer counseling

Using the Claus Model, we identified 18 individuals (18/499 = 3.6 %) who had a greater than 20 % lifetime risk of breast cancer (based on family history) and would therefore be candidates for high risk screening according to ACS guidelines. 5 of these 18 (28 %) individuals were identified using only first degree family information (Table 1).

**Table 1:** Numbers of individuals in each group who met criteria for high risk cancer screening and/or referral for genetic counseling and testing

	Total identified # and % of total cohort	1^st^ degree	2^nd^ degree
Screening			
Breast cancer	18 (3.6 %)	5	13
Colon cancer	62 (12 %)	50	12
Referral for GC/GT			
Breast cancer	9 (1.8 %)	7	2
Colon cancer	31 (6.2 %)	10	21

Using the RST, we identified 9 individuals (9/499 = 1.8 %) who would be candidates for referral for hereditary breast cancer counseling. 50 % as many women who met criteria for high risk breast cancer screening met criteria for high risk breast cancer genetic referral. Using only first degree family information, 7 of these 9 (77 %) individuals would be candidates for genetics referral.

### Identification of candidates for high risk colon cancer screening or referral for hereditary colon cancer counseling

Using the ACS guidelines for high risk colon cancer screening, we identified 62 individuals (62/499 = 12 %) who would be candidates for high risk colon cancer screening. Using only first degree information, 50 of these 62 (81 %) individuals would be candidates for high risk screening (Table 1).

Using modified Amsterdam II criteria we identified 31 candidates (31/499 = 6.2 %) for referral for screening for hereditary colon cancer. 50 % of women who met criteria for colon cancer genetics referral met criteria for high risk colon cancer screening. Using just a first degree family history, 10 of 31 (32 %) of these individuals would be candidates for referral.

## Discussion

**Fig. 2:**
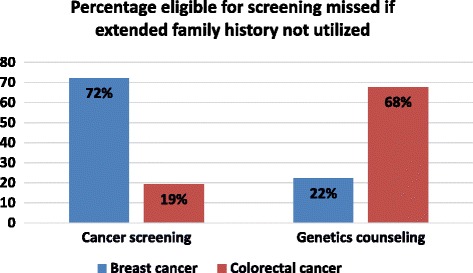
Graph depicting the percentage eligible for screening that would be missed if an extended family history was not utilized

To our knowledge, this is the first study evaluating the added value of an extended family history (vs limited or 1^st^ degree only) using current professional society cancer screening guidelines. Using guidelines from the ACS, 72 % of candidates for high risk breast cancer screening and 19 % of candidates for high risk colon cancer screening would have been excluded if extended (2^nd^ degree) family history was not taken into account (Fig. 2). Additionally, taking only a first degree family history would have failed to identify 22 and 67 % of candidates for referral for genetic counseling for hereditary breast or colon cancer, respectively (using the RST and Amsterdam II criteria) (Fig. 2).

While the gold standard pedigree is a five generation pedigree, this can take over one hour to obtain and is not practical in the primary care setting. Many primary care providers take only a first degree family history. In surveys conducted, less than half of primary care providers and only 65 % of oncologists reported always obtaining a second degree family history [11, 20].

We have shown that taking such a limited family history (1^st^ degree relatives only) would fail to identify a significant number of individuals who would be candidates for high risk cancer screening or referral for cancer genetic counseling. This supports ASCO’s recent recommendations that a minimum family history for cancer patients include 1^st^ and 2^nd^ degree family history information [9].

Our study does have limitations that must be considered. All study participants were female and of predominantly Caucasian background. Family histories were obtained from women reporting for mammography centers creating potential selection bias as women with a positive breast cancer family history are at increased likelihood to be up-to-date for mammography screening [20]. Our family history data was obtained in 2001–2002; however, the technology for taking a family history has not changed. In fact, we still use this same questionnaire in clinic today.

## Conclusions

We have shown that a significant proportion of candidates for high risk breast and colorectal cancer screening and genetic referral would be excluded if family history is limited to first degree family history. Our data set is small and these conclusions should be validated in a larger and more diverse data set.

## Additional file

Additional file 1: Family History Questionnaire. (PDF 27 kb)

## Abbreviations

ACoSOG: American College of Obstetrics and Gynecology
ACS: American Cancer Society
ASCO: American Society of Clinical Oncology
HNPCC: Hereditary nonpolyposis colorectal cancer
NCCN: National Comprehensive Cancer Network
RST: Referral screening tool
SERM: Selective estrogen receptor modulators
USPSTF: US Preventive Service Task Force
UVMMC: University of Vermont Medical Center
