# Supplementary material for: Contribution of extended family history in assessment of risk for breast and colon cancer
Source: BMC Fam Pract. 2016 Sep 1;17(1):126. doi: 10.1186/s12875-016-0521-0 (PMC5007707; doi:10.1186/s12875-016-0521-0)
Supplement: Additional file 1: — Family History Questionnaire. (PDF 27 kb) [file 12875_2016_521_MOESM1_ESM.pdf]

FCP# :__________

Family History Questionnaire
Familial Cancer Program Clinic

Most people have a history of cancer in their families. This is because 1 in 3 people will develop cancer in their lifetime. Familial cancers may be due to inherited genes, a shared environment, or chance. Filling out the following questionnaire will help determine if inherited factors may be playing a role in the development of cancer in your family. Please also list other medical conditions that your relatives have which you feel would be important to know about.

Your name____ Date of Birth__________________

Address____ Telephone number ___________________

____

Have you ever been diagnosed with cancer? ( ) Yes ( ) No

If yes, what type(s) of cancer? _______________________________________

At what age(s) were you diagnosed? _________________________

Family History: Please note whether relatives are living or deceased (L or D)

Your Immediate Family

	**Total Number**	**Number with Cancer**	**Type(s) of cancer**	**Age(s) at diagnosis**
Your daughter(s)				
Your son(s)				
Your brother(s)				
Your sister(s)				

Your mother's side of the family

	Total Number	Number With Cancer	Type(s) of Cancer	*Age(s) At Diagnosis*
Your mother				
Your mother's mother				
Your mother's father				
Your mother's brother(s)				
Your mother's sister(s)				
Your maternal cousins (first cousins)				

What is your mothers ethnic background (country of origin?) ______________
(i.e. English, Irish, French Canadian, African American, Eastern European, Jewish, etc.)

FCP# ___________

Your father's side of the family

	Total Number	Number With Cancer	Type(s) of Cancer	*Age(s) At Diagnosis*
Your father				
Your father's mother				
Your father's father				
Your father's brother(s)				
Your father's sister(s)				
Your paternal cousins (first cousins)				

What is your fathers ethnic background (country of origin?) ______________
(i.e. English, Irish, French Canadian, African American, Eastern European, Jewish, etc.)

Other relatives:

This questionnaire asked you about those relatives most closely related to you. If you have other more distantly related relatives that have been diagnosed with cancer, or have a medial condition you feel would be of importance to us, please list them below. In particular, include those relatives that have been diagnosed with cancer at a young age and/or have been diagnosed with more than one type of cancer.

**Relative's relationship to you (e.g. niece, nephew, maternal, paternal, etc.)**	**Type(s) of cancer**	**Age(s) at diagnosis**

Have you, or any of your relatives ever had genetic testing or participated in a genetic research study? If yes, please explain.

Feel free to add any comments and/or other family medical history you feel would be relevant.

Thank you for taking the time to fill out this questionnaire! All information is kept confidential.

Please return the questionnaire to:

Wendy McKinnon, M.S.
Genetic Counselor
Familial Cancer Program
1 Mill Street, Box B-10
Burlington, VT 05401
(802) 658-4310
